# Leveraging Polymorphism in YbCuBi to Map Transport and Elastic Properties

**DOI:** 10.1021/acs.chemmater.5c02867

**Published:** 2026-01-26

**Authors:** A. K. M. Ashiquzzaman Shawon, George Yumnam, Hsin Wang, Qiang Zhang, Douglas L. Abernathy, Michael E. Manley, Jose L. Mendoza-Cortes, Raphaël P. Hermann, Alexandra Zevalkink

**Affiliations:** † Department of Chemical Engineering and Materials Science, 3078 Michigan State University, East Lansing, Michigan 48824, United States; ‡ Materials Science and Technology Division, 6146 Oak Ridge National Laboratory, Oak Ridge, Tennessee 37831, United States; § Neutron Scattering Division, 6146 Oak Ridge National Laboratory, Oak Ridge, Tennessee 37831, United States; ∥ Department of Physics and Astronomy, Michigan State University, East Lansing, Michigan 48824, United States

## Abstract

*AMX* Zintl compounds with the hexagonal ZrBeSi structure have gained significant attention for their remarkable vacancy tolerance and low thermal conductivity. Their 2D honeycomb sublattice, composed of *M–X* covalent bonds, is believed to contribute to high anharmonicity and unusual thermal transport properties. In this study, we explore the temperature-dependent polymorphism of YbCuBi as a model system to investigate the relationship between the structure and elastic and thermal transport properties in *AMX* Zintls. YbCuBi undergoes a structural transition from the “flat” Cu–Bi layers in the ZrBeSi structure to corrugated layers in the LiGaGe structure below 410 K, resulting in a distortion of its centrosymmetric structure. To probe the effects of this crystallographic transition, we employ inelastic neutron scattering and temperature-dependent resonant ultrasound spectroscopy. These experimental findings, coupled with first-principles calculations and thermal conductivity measurements, allow us to elucidate a direct relationship between corrugation of the honeycomb lattice and the observed changes in elastic and thermal transport properties. These insights can be extended to other Zintl phases with similar structure types, providing a platform for the rational design of functional materials with tailored thermal properties.

## Graphical abstract

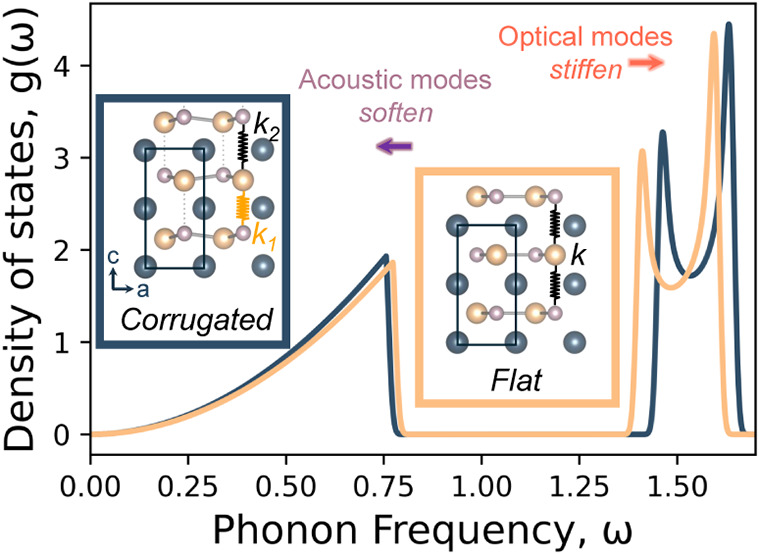

## 1 Introduction

Thermal management has garnered significant attention in recent years, particularly in the energy and electronics industries.^1^ As devices become more powerful and compact, efficient heat dissipation becomes critical to maintaining performance and preventing overheating. Consequently, the ability to tune the thermal properties of materials has become paramount for optimizing device functionality and reliability. Heat flow density (*J*_heat_) within a material is governed by Fourier’s law, expressed as *J*_heat_ *= −*κ × |∇*T*|, where ∇*T* is the temperature gradient and κ represents the thermal conductivity—a measure of a material’s ability to conduct heat.^2^ In solids, the majority of heat conduction occurs via two primary mechanisms: mobile electrons (κ_E_) and lattice vibrations or phonons (κ_L_),^3^ leading to κ = κ_E_ + κ_L_.^4^ In semiconductors and insulators, understanding the factors affecting the phonon contribution is essential for tailoring the thermal properties. Within the Debye–Callaway model, the lattice thermal conductivity is expressed as

1 $$\kappa_{L}=\frac{1}{3}\int C\left(\omega \right)\cdot {\left(v_{g}\left(\omega \right)\right)}^{2}\cdot \tau \left(\omega \right)d\omega$$

where *C*(ω) is the spectral heat capacity, *v*_g_(ω) is the spectral group velocity of vibrational modes, and τ­(ω) is the spectral phonon relaxation time between scattering events.^5^ The spectral heat capacity depends on the phonon density of states *g*(ω), representing the number of available vibrational modes at each frequency, and the Bose–Einstein thermal occupation factor. Acoustic phonon modes, characterized by low energy and high velocity, typically dominate thermal transport due to their larger *v*_g_, longer τ, and higher thermal occupation factors. The velocity is related to a material’s elastic moduli (*c*_*ij*_) and density (ϱ) through the relation *v* ^2^ ∝ *c*_*ij*_/ϱ.^6,7^ By manipulation of these parameters, the thermal conductivity of a material can be effectively tailored to targeted applications.

While the dependence of κ_L_ on phonon modes is well established, the direct influence of the bonding behavior and crystal structure on κ_L_ remains less clearly understood. Zintl compounds, which crystallize in a diverse array of structure types controlled by valence electron count and chemical factors such as ionic radius and electronegativity, offer an exceptional opportunity to investigate this interplay. These compounds also feature inherently low lattice thermal conductivity and tunable electronic properties, making them exciting candidates for applications like thermoelectricity.^8–10^ The *AMX* (*A* = alkaline-earth/rare-earth metals, *M* = transition metals, *X* = metalloids) family of Zintl compounds, in particular, forms a series of closely related structures exhibiting varying degrees of covalent bonding dimensionality.^11,12^ The most prominent is the ZrBeSi-type structure (space group: *P6_3_/mmc*), characterized by planar hexagonal *M–X* nets, separated by *A*-site cations, which occupy 12-fold coordinated hexagonal prisms. While the in-plane *M–X* bonds are generally polar-covalent in nature, the out-of-plane (*M,X*)*−A* interactions are primarily ionic (Figure 1).^13^ Several promising thermoelectric materials have recently been discovered to crystallize in the ZrBeSi-structure type, as shown in Figure 1.^14–16^ The planar *M–X* layers are thought to be responsible for high electronic mobility^17^ as well as, in some instances, soft, anharmonic phonon modes.^14^ The latter phenomenon has been linked to large, out-of-plane atomic displacement parameters (ADP) on the *M* site, a characteristic that has been observed experimentally, most notably on the Ag site in SrAgBi^18^ and on the Cu site in YbCuBi.^19^ While the large ADPs may indeed have a phononic origin, it is also possible that they represent local disorder on the *M* site, a possibility that has not been thoroughly investigated. The planar honeycomb sublattice of the ZrBeSi structure has also been linked to other emergent phenomena, like frustrated magnetism,^20^ hidden spin–orbit coupling,^21^ and the possible discovery of Majorana fermions.^22^

**Figure 1:**
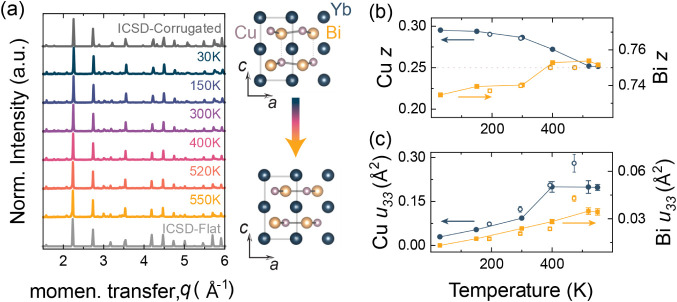
(a) Neutron powder diffraction is shown as a function of temperature as YbCuBi transitions from the corrugated to flat structure. (b) Cu (Bi) *z-*positions, as extracted from Rietveld refinement of NPD, show a systematic change with increasing temperature toward higher symmetry, reaching a value of 0.25 (0.75) above 400 K. (c) The smaller Cu atom displays large *z-*atomic displacement parameters compared to the larger Bi atom. The results are consistent with a crystallographic phase transition from the low-temperature corrugated structure to the high-temperature “flat” structure at 410 K. Open symbols represents data taken from ref 19.

Although the planar honeycomb *M–X* lattice is frequently cited for its interesting electronic and phonon behavior, the relationship between the honeycomb *M–X* layers and transport properties has remained elusive. The LiGaGe-type structure (also referred to as LiZnSb-type in the literature) is closely related to the ZrBeSi structure, except in one critical way—the honeycomb lattice exhibits out-of-plane corrugation. As shown in Figure 1, every alternating atom is periodically displaced along the *c*-axis, similar to an accordion. Electron localization function (ELF) analysis from density functional theory (DFT) has shown interplanar interactions emerging between neighboring *M–X* layers in some compounds with the LiGaGe-structure type, suggesting a shift toward 3-dimensional covalent bonding.^11,23^ Computational studies have suggested that the dimensionality of the covalent sublattice in *AMX* compounds may strongly impact elastic properties and, therefore, thermal properties. However, such relationships have never been proven experimentally.^11,24^ Here, we investigate YbCuBi, which undergoes a *corrugated-to-flat* transition upon heating above 410 K,^19,25^ as a model material to study the role of *M–X* layer buckling on thermal and elastic properties. We employed a combination of computations and experiments to elucidate the impacts of structural corrugation on bulk elasticity and thermal conductivity. By combining two complementary techniques, inelastic neutron scattering (INS) and resonant ultrasound spectroscopy (RUS), we were able to show that the planar-to-corrugated phase transition has a very different impact on acoustic phonons compared to optical phonons.

## 2 Experimental and Computational Methods

### 2.1 Computation

First-principles calculations were carried out within the DFT framework using the Vienna *ab initio* simulations package (VASP).^26^ Generalized gradient approximation-based Perdew–Burke–Ernzerhof (PBE-GGA) exchange–correlation functionals and projector-augmented wave (PAW) pseudopotentials were used in these calculations.^27^ The low-temperature LiGaGe (193 K) and high-temperature ZrBeSi structures (473 K) were collected from ICSD, as reported on single crystals by Tkachuk et al.^19^ A converged *k-*point mesh of 11 × 11 × 6 and an energy cutoff of 500 eV were used to relax the crystal structure and reach self-consistency. The elastic tensors were calculated from the relaxed structure using stress–strain relationships.^28^ A 3 × 3 × 2 supercell was used in this calculation with a *k*-point sampling of 4 × 4 × 4. The phonopy package was used to calculate the force constants via the finite displacement method.^29^ The results were used to extract phonon band structures, density of states, and the partial density of states.

### 2.2 Synthesis

Bulk polycrystalline samples of YbCuBi were synthesized by using a powder metallurgical route. Stoichiometric amounts of Bi shots (5N plus, 99.999% purity) were weighed into a stainless-steel SPEX vial inside an argon-filled glovebox. Bi was ball-milled with three 7/16″-stainless steel ball bearings for 10–15 min to create an inner coating on the vial walls. This procedure is maintained throughout all samples to prevent ductile metals from sticking to the vial walls. Then, Yb sheets (Edgetech Industries, 99.99% purity) were surface-polished and cut into sub-millimeter-sized pieces. Cu powder (Alfa Aesar, 99.9% purity) and cut Yb were then added to the SPEX vial with premilled Bi inside the glovebox. The mixture was ball-milled for 1 h, scraped using a stainless steel spatula inside the glovebox, and then ball-milled again for another hour. This process ensures thorough and homogeneous mixing. The resultant powders were then scraped out of the vial inside an Ar-filled glovebox. The powders were subsequently sintered inside 10 mm diameter graphite dies. Spark plasma sintering (SPS) was done at 1073 K for 10 min under 50 MPa uniaxial pressure to obtain dense pucks with a relative density >94%. These pucks were then used for structural, elastic, and thermal transport properties characterization. The sintered puck was found to be stable in air for at least several days at ambient temperature. For neutron diffraction and inelastic neutron scattering measurements, a large amount of sample is required to obtain sufficient statistics. Therefore, multiple batches of YbCuBi pucks were synthesized using ball milling and SPS. These pucks were then crushed using an alumina mortar–pestle and mixed. Approximately 6.5 g of the resultant powders was wrapped in graphite foil and sealed inside an evacuated quartz tube with a small positive pressure of argon. The powders were then annealed at 550 K for 3 days to improve crystallinity and homogeneity.

### 2.3 Structural Characterization

SPS’d polycrystalline pucks were polished and characterized using X-ray diffraction (XRD) with a Rigaku SMARTLAB diffractometer equipped with a Cu–Kα radiation source. Lattice parameters were calculated through Rietveld refinement (RR) using the Rigaku PDXL-2 software. Annealed powders were also characterized using XRD. Neutron powder diffraction (NPD) was carried out at the BL-11A (POWGEN) beamline of the Spallation Neutron Source (SNS), Oak Ridge National Laboratory (ORNL). An 8 mm diameter vanadium can was loaded with ∼6.3 g of YbCuBi powders and fitted with a titanium lid. The filled can was put inside a closed-cycle Janis cryo-furnace and evacuated for the scattering experiment. Measurements below room temperature (300 K) were conducted under a low partial pressure of He gas, while high-temperature (>300 K) measurements were conducted under a vacuum atmosphere. A neutron frame with a center wavelength of 0.8 Å was used for data collection at multiple temperatures below and above the transition temperature (*T*_c_). Rietveld refinement of neutron diffraction results was performed using the GSAS-II software.^30^

### 2.4 Elastic and Transport Properties

RUS, a nondestructive characterization technique, was employed to measure the resonant frequencies of dense SPS’d pellets.^31^ Each peak in the resonant spectra represents a vibrational mode with a wavelength on the order of the specimen size. The open-source RUSpy software was used to collect the spectra. The room-temperature spectra were collected using the 3D-printed setup from Alamo Creek Engineering (ACE),^32^ while the high-temperature spectra were collected on the custom-built HT-RUS setup from ACE. The RUScal software was used to extract the elastic constants by inverse numerical analysis, where 40–52 resonance peaks were fit at all temperatures.^33^ The isotropic model was sufficient to model two independent elastic constants, *c*_11_ and *c*_44_, since the samples were bulk polycrystalline with randomly oriented grains. The elastic constants were used to calculate sound velocities and elastic moduli.^7^ Thermal diffusivity was measured by using a NETZSCH Light Flash Apparatus (LFA) 467 HyperFlash. The equation κ *=* ϱ × *D* × *C*_p_ was used to calculate the total thermal conductivity, κ, where ϱ is the geometric density, *D* is thermal diffusivity, and *C*_p_ is the specific heat capacity that was estimated using the Dulong–Petit approximation. Specific heat capacity was also measured on the sintered pucks (mass, 180.86 mg) using differential scanning calorimetry (DSC; NETZSCH DSC 404 F1). Electrical resistivity and Seebeck coefficients were measured using cylindrical samples of dimensions ∼3 × 2 × 9 mm^3^. The measurements were conducted using a ULVAC ZEM-III instrument under a positive pressure of He gas. The electrical resistivity, ρ, was used to calculate κ_E_ using the Wiedemann–Franz (WF) Law, κ_E_ *= L*·*T*/ρ, where *L* is the Lorenz proportionality constant.^34^ κ_L_ was calculated by using the simple relationship, κ_L_ = κ – κ_E_.

### 2.5 Inelastic Neutron Scattering

INS measurements were conducted at the BL-18 (ARCS) beamline of the SNS. A 6 mm diameter Al can was filled with the powder sample, closed with a Ti lid, and loaded into a Janis CCR stage with an automatic sample changer. The INS spectra of ∼6.69 g of YbCuBi powders were measured for 2 h at each temperature using Powgen Automatic Changer (PAC) cans, with an empty can recorded for background subtraction for sufficient scattering statistics. INS measurements were conducted at *T* = 30, 150, 300, 400, and 520 K. An incident energy of 45 meV was used. Data reduction from instrument coordinates to the scattering function (S­(E)) was performed using the MANTID software.^35^ S­(E) transformation to the density of states (DOS) and multiphonon corrections were performed using a modified version of the DOS code.^36^

## 3 Results and Discussion

### 3.1 Structure Characterization

Rietveld refinement of the neutron powder diffraction (NPD) pattern at 30 K reveals that YbCuBi makes up 90.6 atom % of the bulk powder, while secondary phases Cu, Bi, and Yb_2_O_3_ make up the rest (see Figure S2). Note that NPD measurements were collected on annealed samples, which were necessary to improve crystallinity (detailed descriptions are provided in SI Section 2). NPD collected as a function of temperature is shown in Figure 1a. The temperature-dependent lattice parameters, extracted through Rietveld refinement, are shown in Figure S3a. The phase transition in YbCuBi is not associated with an abrupt change in lattice parameters, and as a result, the diffraction patterns show only minor changes as a function of temperature. These results qualitatively agree with the lattice parameters reported by Tkachuk et al. but differ from the results presented by Merlo et al.^19,25^ A narrow range of momentum transfer was selected for a zoomed-in inset in Figure S3b, where the temperature evolution of two peaks is shown. With increasing temperature, the (101) peak loses intensity but does not completely disappear. This is consistent with the results of Tkachuk et al., where the X-ray diffraction data were collected on single crystals.^19^ The integrated intensity (area) of the (101) peaks is shown in Figure S1c, which is directly proportional to the square of the structure factor (*I* ∝ |*F*_101_|^2^). The intensity decreases with increasing temperature and reaches a near-zero plateau above 400 K, which is indicative of the crystallographic transition. The *z-*atomic positions and ADPs along *z*-direction are shown in Figure 1b and c, respectively. Symmetry constraints restrict the *x*- and *y*-positions of Cu (Bi) atom(s), while the deviation of the *z-*position from 0.25 (0.75) represents the degree of corrugation. We can see a gradual change in *z-*position as a function of temperature as the corrugation disappears above 400 K. Consistent with prior reports of Tkachuk et al., the Cu atoms display significantly higher ADPs in comparison to the Bi atoms, which increase with temperature until the phase transition.^19^

### 3.2 Specific Heat Capacity and Transport Properties

Thermal conductivity is typically experimentally measured as a product of heat capacity, thermal diffusivity, and density. In this study, we measured the thermal flow using DSC (on powder samples), specific heat capacity (on a polycrystalline puck) and thermal diffusivities (on a polycrystalline puck) independently, as shown in Figure S4. The calorimetry profile on heating does not show evidence of the corrugated-to-flat phase transition, likely due to fast temperature scans. A large, reversible peak is also observed above 510 K, likely due to secondary phase (Bi) melting. On cooling, a peak arising from the crystallographic phase transition with an onset temperature of 410 K is observed. It is worth noting that the crystallographic phase transition is observed ∼40 K higher than the reports from both Tkachuk and Merlo et al.^13,19,25^ Furthermore, an additional peak is observed at ∼470 K in the specific heat capacity, which was not reproducible in DSC using powder samples and was not accompanied by anomalies in thermal diffusivity, RUS, or electrical conductivity. The measured specific heat capacity of 0.248 J g^–1^ K^–1^ (110.5 J mol^–1^ K^–1^) was found to be 50% higher than that calculated from the Dulong–Petit approximation (*C*_p_ = *C*_v_ = 3*R*). As other authors have shown, specific heat capacity measurements are extremely susceptible to experimental error and represent a large source of uncertainty in determining κ.^37^ Therefore, for consistency across the phase transition and heating profiles, the Dulong–Petit heat capacity was used to calculate the thermal conductivity. It is worth noting, however, that the Dulong–Petit approximation does not account for the latent heat of a phase transition or changes in volume due to thermal expansion.^7,38^

On the other hand, thermal diffusivity measurements within the laser-flash method are more reproducible. At the phase transition temperature, a step-like decrease is seen in thermal diffusivity on cooling. Finer temperature steps on heating confirmed a 5% *increase* in thermal diffusivity, which is reversible upon cooling. Furthermore, the phase transition temperature is consistent with the differential scanning calorimetry (DSC) scans and the NPD results. Assuming constant heat capacity (Dulong–Petit approximation) and constant density, the total thermal conductivity is calculated as shown in Figure 2a. To decouple the contributions of lattice vibrations and mobile charge carriers to thermal conductivity, we calculated the electronic thermal conductivity using the Wiedemann–Franz approximation, κ_E_ *= L*·*T*/ρ. The electrical resistivity, ρ, is shown in Figure S4c. The resistivity and Seebeck coefficient show no sharp discontinuity in the temperature range of the phase transition, in contrast to Merlo et al.^25^ The broad features in slope/curvature (Figure S4c inset) exhibit magnitudes that are small compared to the uncertainties in the resistivity measurement using ZEM-III. Any coupling between structural transition and electronic properties appears weak within the accuracy of the present measurements. Note that the Seebeck coefficient is negative, suggesting that electrons are the majority charge carriers.^38^ Both electronic properties are indicative of highly degenerate semiconducting behavior; therefore, we employ the degenerate limit of the Lorenz number to calculate κ_E_.^39^ Lattice thermal conductivity, κ_L_, was calculated by subtracting κ_E_ from total κ. As seen in Figure 2a, κ_L_ exhibits a 6% step-like change associated with the crystallographic phase transition.

**Figure 2:**
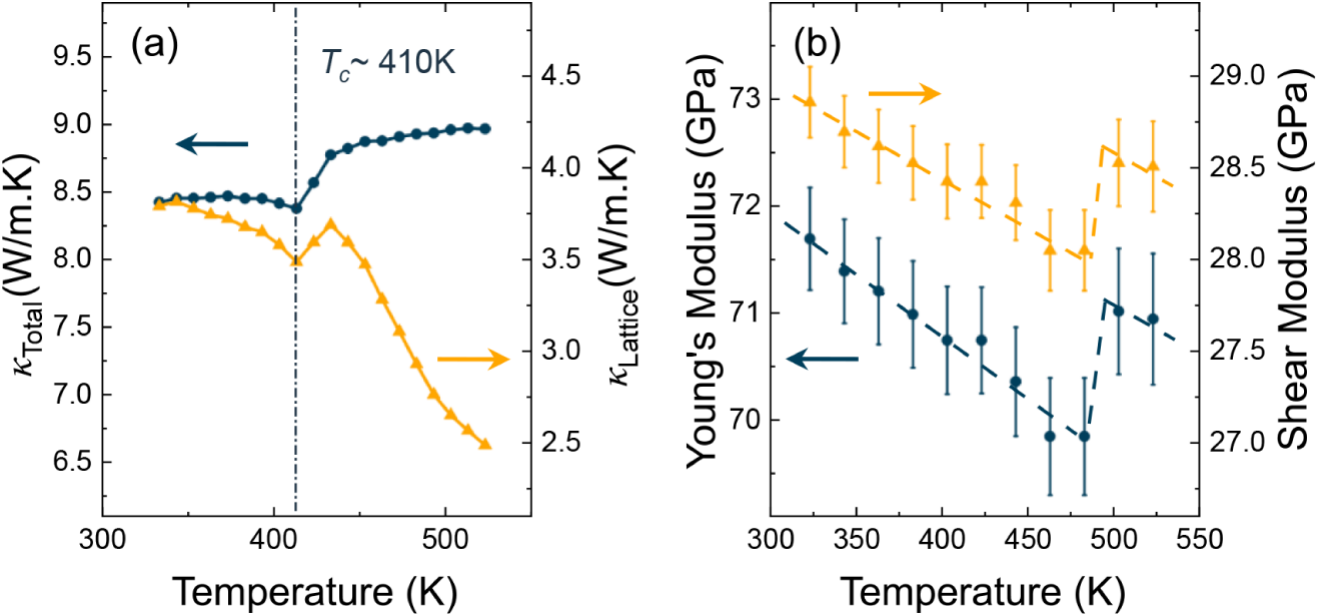
(a) Total thermal conductivity, κ_Total_, exhibits a step-like increase as the crystal structure changes from corrugated-to-flat in YbCuBi. Lattice thermal conductivity, κ_lattice_, shows a 6% increase. (b) The phase transition is also accompanied by an increase in Young’s and shear modulus, which suggests stiffening of acoustic phonon modes.

Thermal diffusivity was measured on the sintered pucks, before the secondary powder annealing step introduced quantifiable secondary phases (see Figure S2, top panel). Commercially available laser-flash measurements can introduce ∼3% uncertainty, while additional errors may arise from the Dulong–Petit approximation (∼5%) and density measurements (∼1%).^40^ Consequently, absolute values of thermal conductivity and lattice thermal conductivity may carry an overall 5–6% uncertainty. Therefore, the absolute values of thermal conductivity should be treated with appropriate caution. However, the reversible trend observed in thermal diffusivity holds true, with the *corrugated-to-flat* transition exhibiting a *step-like increase* in total and lattice thermal conductivities.

### 3.3 Phonon Behavior

The decrease in κ_L_ on cooling at the phase transition warrants further investigation of the underlying mechanism. To assess changes in phonon velocity across the entire frequency spectrum, we combined speed-of-sound measurements, which measure acoustic phonons, with inelastic neutron scattering, which spans the full frequency range but offers less detail for the acoustic modes. The speed of sound was measured using resonant ultrasound spectroscopy (RUS). As the YbCuBi sample is heated, bonds become softer due to thermal expansion, which leads to a nearly linear reduction in resonant frequencies.^41^ Figure S5 shows the temperature evolution of two of the resonance peaks. At the phase transition, the resonant frequencies abruptly increase, representing a step-like stiffening mechanism. The temperature-dependent Young’s and shear moduli, obtained from the resonant modes by inverse numerical analysis, also show a small ∼2% stiffening at the phase transition, as shown in Figure 2b. Above the phase transition, further heating leads to continued softening. No thermal hysteresis was observed on cooling. Note that the temperature is overstated by 50–60 K in this measurement due to the position of the sample thermocouple at a hotter location in the furnace.^41^

To aid in the analysis of inelastic neutron scattering (INS) measurements, the phonon band structure and density of states were calculated from first-principles using DFT for the low-temperature LiGaGe structure (Figure S6 and Figure 3a). Note that 0 K DFT calculations for the high-temperature structure types produced imaginary phonon branches, suggesting that the structure is dynamically unstable. Therefore, only the results for the LiGaGe-structure type are used in our analysis of the INS data. The partial density of states shows that all three elements contribute to the acoustic phonons. Typically, light atoms participate more in high-frequency optical modes, while heavy atoms participate more in acoustic phonons. In this case, however, the lighter Cu atoms display low bond stiffness, thereby contributing substantially to the lower-energy acoustic modes. The prior reports of Cu atoms having large ADPs in the LiGaGe structure would be consistent with Cu having very low bond stiffness, particularly in the out-of-plane direction.^19^ Similar behavior has been previously observed in trigonal planar-coordinated Cu atoms.^42,43^ Nonetheless, light Cu atoms also dominate the high-energy optical phonons above the band gap of ∼16 meV.

**Figure 3:**
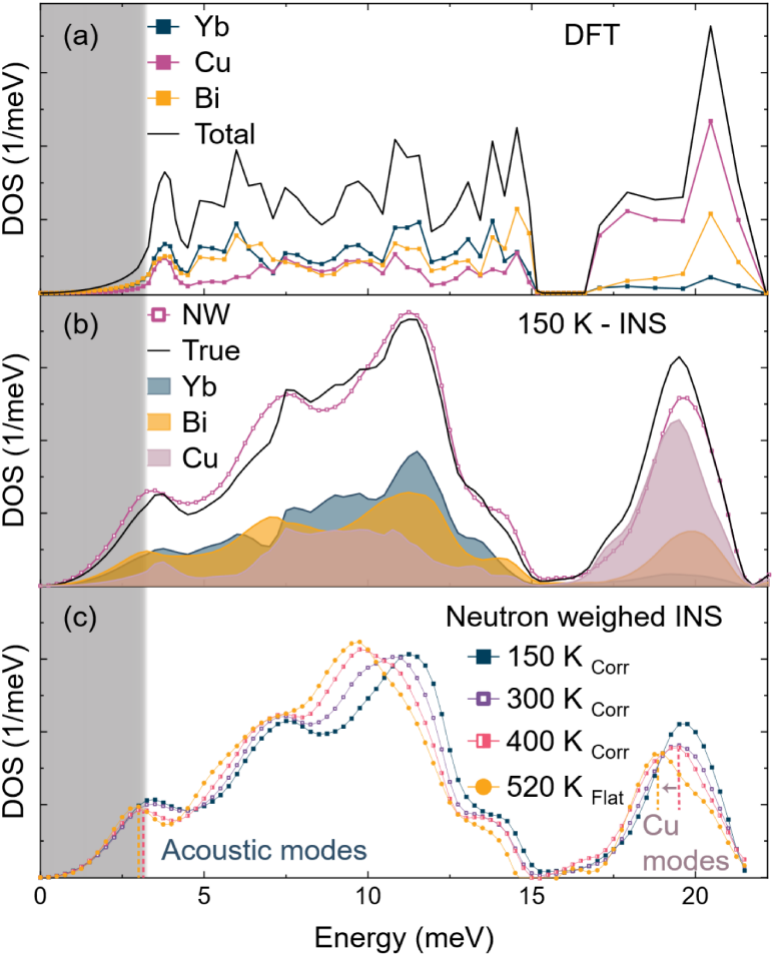
(a) Phonon density of states was calculated from DFT for the corrugated YbCuBi structure. Partial DOS shows that the light Cu contributes to acoustic phonons, while heavy Bi modes are also associated with high-energy optical phonons. (b) Using the partial DOS from DFT, the experimental INS spectra can be resolved into element-specific partial DOS, after adjusting for neutron scattering corrections. (c) INS spectra reveal that, at the phase transition, the Cu-optical modes show significant softening.

Inelastic neutron scattering measurements were conducted on the polycrystalline powders to assess the behavior of phonons across the phase transition. The neutron scattering cross-section is different for each element, and the ratio of neutron scattering cross-section (σ_*i*_) and nuclear mass (*m*_*i*_) determines how neutrons interact with a given element.^44^ The neutron-weighting factors for Yb and Cu are ∼3 times that of Bi (σ_Yb_/*m*_Yb_ = 0.135 barns/amu, σ_Cu_/*m*_Cu_ = 0.126 barns/amu, σ_Bi_/*m*_Bi_ = 0.0438 barns/amu). Therefore, the neutron-weighted INS spectra (NW-DOS) are heavily skewed toward Yb and Cu, while Bi contributions are underrepresented. True DOS and element-specific partial DOS were extracted by deweighting the neutron scattering cross-section factors, as shown in Figure 3b. This procedure is detailed in SI Section 6 and in ref 44. Note that the DFT partial DOS used in this analysis was taken from the corrugated structure at all temperatures due to negative phonon modes found in the flat YbCuBi structure.

The NW-DOS collected from INS as a function of temperature is shown in Figure 3c. Within the corrugated structure (below 410 K), Yb-dominated optical phonons, which peak at about 12 meV at 150 K, show large thermal softening on heating. Large temperature-dependent softening of vibrational modes is typically associated with strong anharmonicity.^45^ Moderate softening is also observed in the acoustic modes as the temperature increases from 150 to 400 K, which is consistent with RUS results at temperatures below the phase transition. Interestingly, the Cu/Bi-dominated high-energy optical modes, which peak at approximately 19 meV, exhibit minimal softening in this temperature range. Considering the softening behavior in the lower-energy optical modes, this observation is surprising.

Between 400 and 520 K, high-energy optical modes at ∼18 meV exhibit a step-like softening, which can likely be attributed to the crystallographic phase transition, while the low-energy acoustic modes retain their position. It is worth noting that an elastic cutoff energy of 2.7 meV (shaded region) was used to extract the phonon DOS. Therefore, the phonon modes measured by INS below 3 meV should be interpreted with caution. The RUS measurements described earlier serve as the more accurate measurement of the long-wavelength acoustic phonon behavior.^32^ Furthermore, the Yb-dominated midrange optical phonons continue to soften, following the same trend regardless of the phase transition.

Surprisingly, the 0 K force constants derived from the DFT calculations for the corrugated structure type (shown as the horizontal lines in Figure 4a) show that the light Cu atoms exhibit the lowest force constants, while the massive Bi atoms show the largest value. This suggests a significant disparity in the dynamics of Cu and Bi, despite both elements occupying symmetrically identical 3-fold coordinated sites (Wyckoff site *2b*) in the hexagonal lattice. Because Bi is so much larger than Cu, it is the sum of the Yb^2+^ (∼1.2 Å) and Bi^3–^ (∼2 Å) radii that controls the interlayer spacing in this structure type. In Figure 4b and c, we show the Yb coordination environment—a distorted hexagonal prism—and a close-packed representation of the structure, viewed along [110]. Figure 4b shows that by symmetry the Yb–Cu distances and the Yb–Bi distances must be identical (3.1–3.4 Å in the corrugated structure). This distance is *significantly* longer than the sum of the Cu^+^ (∼0.5 Å) and Yb^2+^ (∼1.2 Å) ions. Thus, while both Cu and Bi experience chemical pressure in the *a–b* plane, only Bi experiences significant chemical pressure from Yb along the *c*-axis. In contrast, Cu is relatively unconstrained in the *c*-direction, which would explain the large ADPs observed in neutron diffraction in both the corrugated low-temperature and the flat high-temperature structure types, as well as the soft force constants observed in the present DFT calculations.^19^

**Figure 4:**
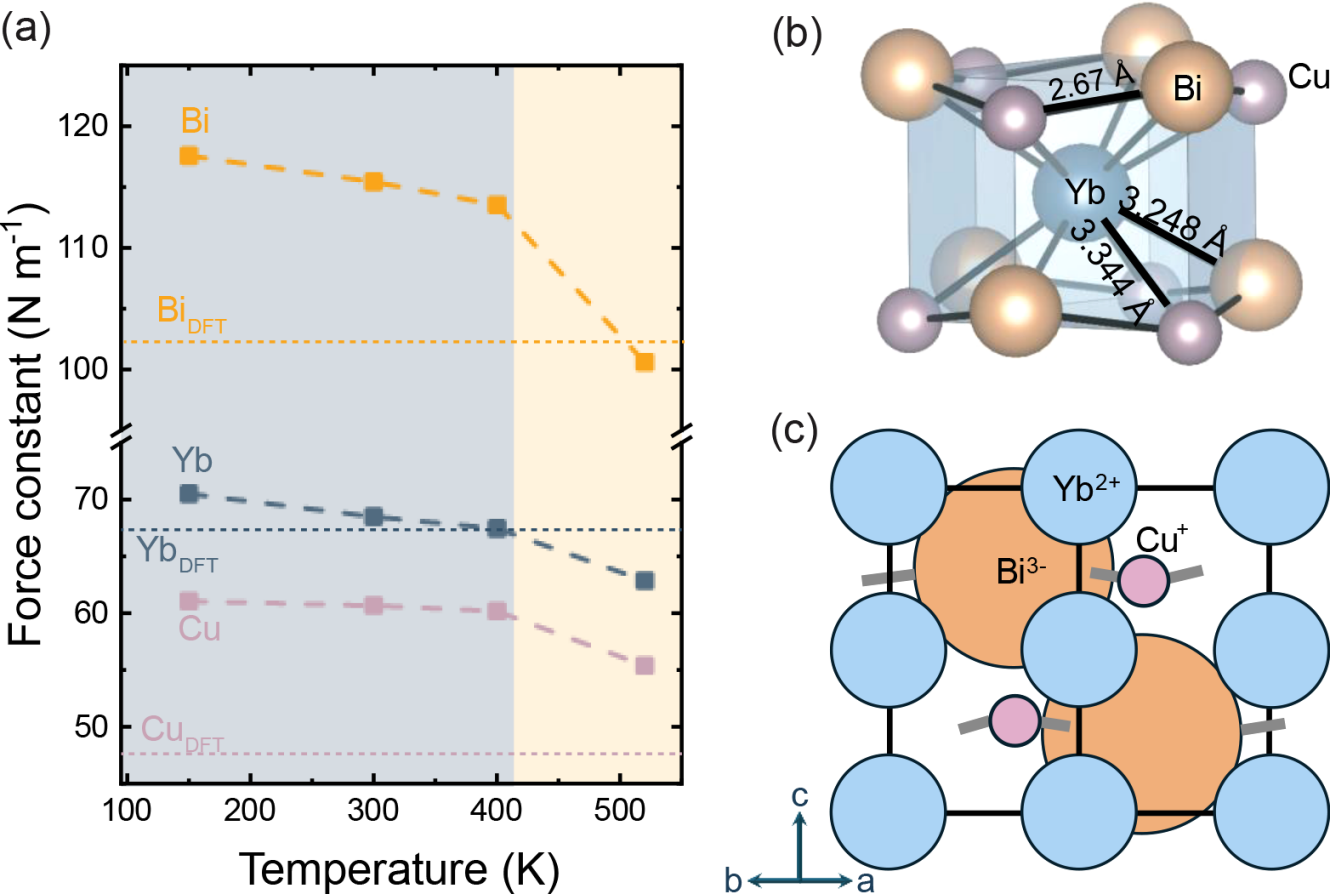
(a) Element-specific force constants were calculated from the experimental partial DOS. (b) In the corrugated structure at 300 K, Yb occupies a distorted hexagonal prism with two unique (Bi,Cu)-Yb bond lengths of 3.248 Å and 3.344 Å. The Cu–Bi distance is 2.67 Å. (c) The close-packed representation assumes ionic radii of *r*_Yb_ ∼ 1.2 Å, *r*_Cu_ ∼ 0.5 Å, and *r*_Bi_ ∼ 2 Å.

The temperature dependence of the force constants shown in Figure 4a was estimated by fitting the experimental DOS using the procedure described in SI Section 6. The element-resolved fit to the experimental DOS at each temperature step is shown in Figure S7. The force constants of all three elements show a significant *decrease* with increasing temperature, a trend that appears to steepen as the crystal structure changes from corrugated-to-flat upon heating. Overall, the INS results are indicative of softer optical phonons in the flat YbCuBi structure, in direct contrast to the RUS results presented in Figure 2b.

### 3.4 Structural Stability and Elastic Tensor from First-Principles DFT

To assess the energy cost associated with the crystallographic transition, DFT was used to calculate single-point energies of YbCuBi using the unrelaxed experimental structures reported by Tkachuk et al. at 193, 295, 393, and 473 K.^19^ The DFT energies are shown as a function of lattice parameter *c* in Figure S1a. The energies are the lowest for the corrugated structure type (193 and 295 K), exhibit a peak for the 393 K structure, and then decrease again slightly at 473 K. This suggests that an energy barrier needs to be overcome before the flat structure can be fully stabilized at larger *c*-lattice parameters (see Figure S1a).

We also turned to DFT in an attempt to understand the observed changes in elasticity caused by the phase transition (Table S1). As input, we used experimental structures from Tkachuk et al. reported at 193 K (corrugated) and 473 K (flat). These were fully relaxed by allowing both lattice parameters and atomic positions to change (the symmetry was conserved). The relaxed structures diverged significantly from the experimental input structures: the corrugated structure was found to contract by 4% in the *c*-direction and expand by 1% in the *a*-direction.^19^ This was accompanied by a change in the Cu–Bi bond angle in the corrugated layers from 117.6° to 113°. The resulting interplanar Cu–Bi distance was 3.03 Å, which is short enough to be considered a primary bond. In contrast, the flat structure type expanded upon relaxation in the *c*-direction ∼1%, while showing minimal change in the a-direction.

Comparing the elastic constants, we find that the relaxed corrugated structure is overall stiffer than the relaxed flat structure. In particular, the elastic constant *c*_33_, which quantifies the out-of-plane stiffness, is 27% lower in the flat structure than in the corrugated structure. This can be attributed to the much larger *c*-lattice parameter structure with “flat” Cu–Bi layers after relaxation (Table S1). Similar results were observed in a prior DFT study of CaAgBi, where a larger *c* leads to decreased *c*_33_.^24^ However, overall, these results are inconsistent with our experimental finding that the elastic moduli become slightly stiffer when YbCuBi undergoes the transition from corrugated to flat. The disparity is likely directly related to the difference between the experimental and relaxed lattice parameters. Note that adding SOC and/or van der Waals interactions does not change the overall trend for lattice parameters or the elastic tensor.

### 3.5 Underlying *Peierls*-like Distortion Mechanism

The *corrugated-to-flat* phase transition in YbCuBi can be characterized as the gain of mirror-plane symmetry perpendicular to the *c*-axis. As observed through RUS and INS, the *gain* of mirror symmetry on heating is accompanied by the simultaneous *stiffening of acoustic phonons* and the *softening of optical phonons*. We can rationalize this phonon behavior using a simple two-spring toy model. Along the *c*-axis, interlayer Cu–Bi bonding can be visualized as two atoms of mass, *m*_1_ and *m*_2_, connected by two springs with spring constants, *k*_1_ and *k*_2_ (Figure 5). As such, we can build a Born–von Kármán two-atom model and solve the dynamical matrix for a column of Cu and Bi atoms stacked along the *c*-direction, as detailed in SI Section 7.

**Figure 5:**
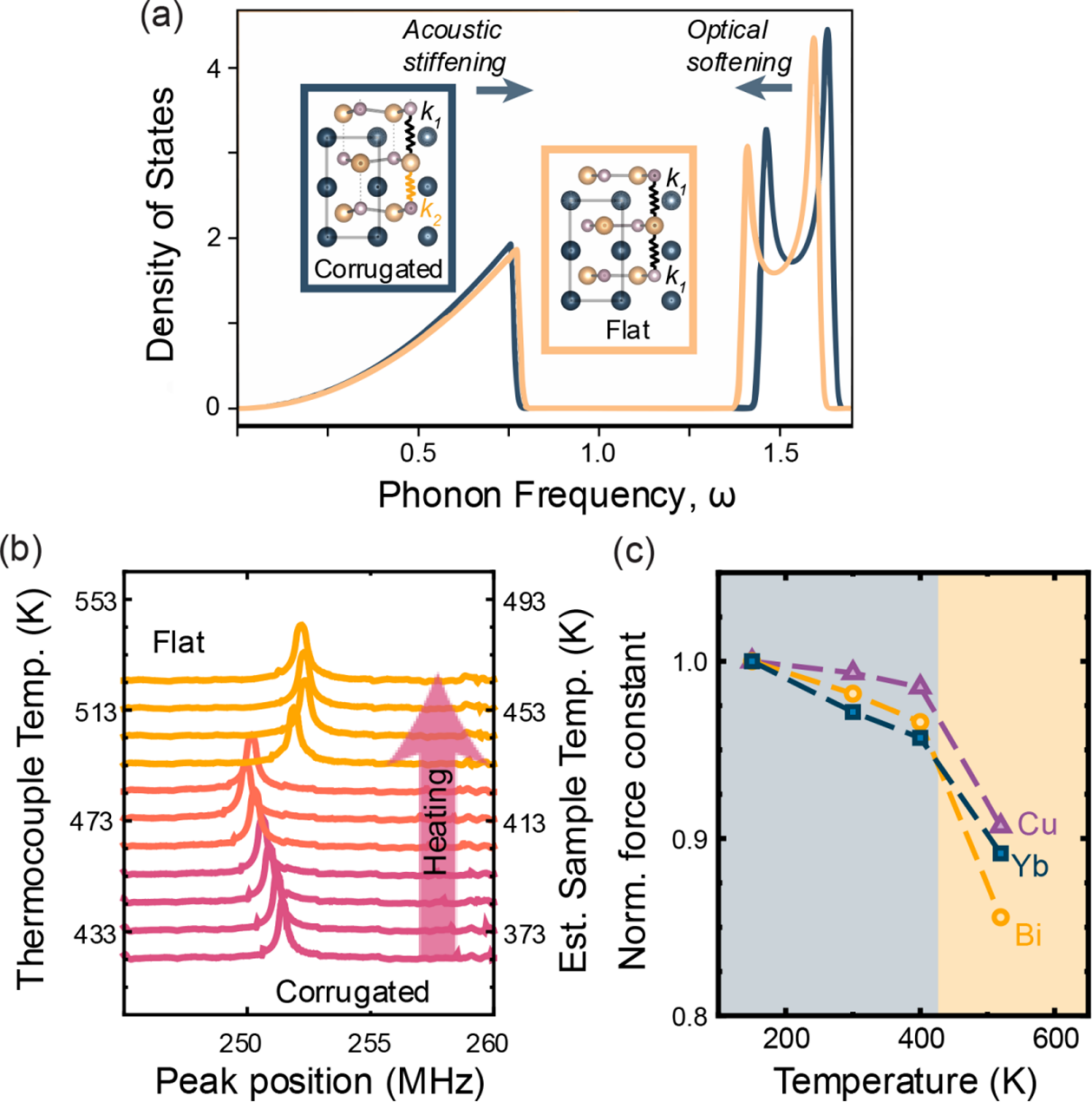
(a) Considering the phase transition to be similar to a *Peierls*-like distortion, the phonon frequencies can be calculated using a simple ball-and-spring model. Solving the dynamical matrix predicts that, at the phase transition, upon *heating*, the acoustic modes would stiffen, while the optical modes would soften. This interpretation is consistent with the experimentally observed results, where (b) RUS shows the stiffening of acoustic modes, while (c) force constants, normalized to force constants at 150 K, from INS show the softening of optical modes. Note that the estimated sample temperature has been calculated with a constant offset of 60 K and may have a 20 K uncertainty.

In the case of YbCuBi, we can compare two hypothetical cases. Within the corrugated structure, the interplanar Cu–Bi bond distances have been reported as ∼3.5 Å and ∼4.5 Å, respectively.^19^ On the other hand, in the flat structure (space group: *P6_3_/mmc*), the Cu–Bi atoms are equidistant, placed ∼4 Å apart. Since attractive forces are proportional to 1/(distance)^2^ (Coulomb’s law), and force is directly proportional to the spring constant (Hooke’s law), we will assume that the force constants *k*_1_ and *k*_2_ take the values of 1/(3.5)^2^ Å^–2^ and 1/(4.5)^2^ Å^–2^ in the corrugated case, while the flat 2D structure exhibits one *k* of value 1/(4)^2^ Å^–2^. We used the respective masses of Cu and Bi, and we assumed an *a*-lattice parameter of ∼6 Å (assuming it remains constant between the two structures).

Solutions to the toy model dynamical matrix, shown in Figure 5, demonstrate that as the flat structure breaks the mirror symmetry, a *Peierls*-like mechanism emerges: acoustic and optical phonon modes push each other in opposite directions. As the corrugated structure turns flat by gaining mirror symmetry on heating, the 1D toy model predicts the *stiffening of acoustic modes* and the *softening of optical* phonon modes. Our combination of temperature-dependent RUS and INS captures this elegant phenomenon in this study. Similar processes are also observed in *Peierls*-like distortions that lead to the formation of charge-density waves and pseudogap formations, both in phonon and electronic band structures.^46^

In the case of YbCuBi, this *Peierls*-like mechanism has a unique consequence as well. High-energy optical phonons behave like standing waves with minimum velocity, while the acoustic phonons exhibit considerable group velocity.^47,48^ As the structure transitions from the *corrugated-to-flat* structure, a velocity increase is observed. Since lattice thermal conductivity correlates with phonon velocity squared, this explains the 6% *increase* observed in κ_L_ despite the softening of optical modes.

## 4 Conclusion

YbCuBi forms the corrugated LiGaGe structure at low temperatures, as seen through neutron diffraction results. Temperature stabilizes the flat ZrBeSi structure above 410 K, as seen using diffraction and calorimetry, where the average structure transforms from corrugated *M–X* layers with weak intralayer interactions into flat, graphene-like honeycomb layers with no interlayer bonding. The phase transition causes an abrupt, step-like increase in lattice thermal conductivity on heating. Resonant ultrasound spectroscopy showed that the acoustic phonons become stiffer in the high-temperature structure, while inelastic neutron scattering observed simultaneous softening of the optical phonons. First-principles calculations combined with a toy model demonstrate that a *Peierls*-like mechanism is responsible for this phonon behavior. We show using a combination of theory and experiments that in YbCuBi, the thermal transport is dominated by the behavior of the acoustic phonons at the phase transition rather than by the optical phonons. This study provides a systematic demonstration of the underlying mechanism behind the thermal conductivity change.

## Supplementary Material





## Data Availability

Raw data from SNS is available at DOI: https://oncat.ornl.gov/dois/68733aa9188444fcfdea605a.

## Notes

Structural stability and phonon band structure from first-principles DFT; X-ray and neutron powder diffraction patterns, temperature-dependent lattice parameters, transport properties, calorimetry, RUS profiles; inelastic neutron scattering data analysis and results; Born–von Kármán model for flat and corrugated structures; (PDF)
ALGORITHM: Phonon Branch pseudocode for 2-spring–2-mass Born–von Karman model (TXT)

## #

Department of Physics, University of Michigan, Ann Arbor, Michigan 48109, USA
