# Supplementary material for: Leveraging Polymorphism in YbCuBi to Map Transport and Elastic Properties
Source: Chem Mater. 2026 Jan 26;38(3):1425–34. doi: 10.1021/acs.chemmater.5c02867 (PMC12895387; doi:10.1021/acs.chemmater.5c02867)
Supplement: Supplementary file 1 [file cm5c02867_si_001.pdf]

Supporting information

Leveraging Polymorphism in YbCuBi to Map Transport and Elastic Properties

A K M Ashiquzzaman Shawon[1,2 †], George Yumnam[2], Hsin Wang[2], Qiang Zhang[3], Douglas L. Abernathy[3], Michael E. Manley[2], Jose L. Mendoza-Cortes[1,4 *], Raphaël P Hermann[2 *], Alexandra Zevalkink[1 *]

[1]Department of Chemical Engineering and Materials Science, Michigan State University, East Lansing, Michigan 48824, USA

[2]Materials Science and Technology Division, Oak Ridge National Laboratory, Oak Ridge, Tennessee 37831, USA

[3]Neutron Scattering Division, Oak Ridge National Laboratory, Oak Ridge, Tennessee 37831, USA

[4]Department of Physics and Astronomy, Michigan State University, East Lansing, Michigan 48824, USA

[†]Current address: Department of Physics, University of Michigan, Ann Arbor, Michigan 48109, USA

*corresponding authors: jmendoza@msu.edu, hermannrp@ornl.gov, alexzev@msu.edu

SI 1. Structural stability from first-principles DFT:

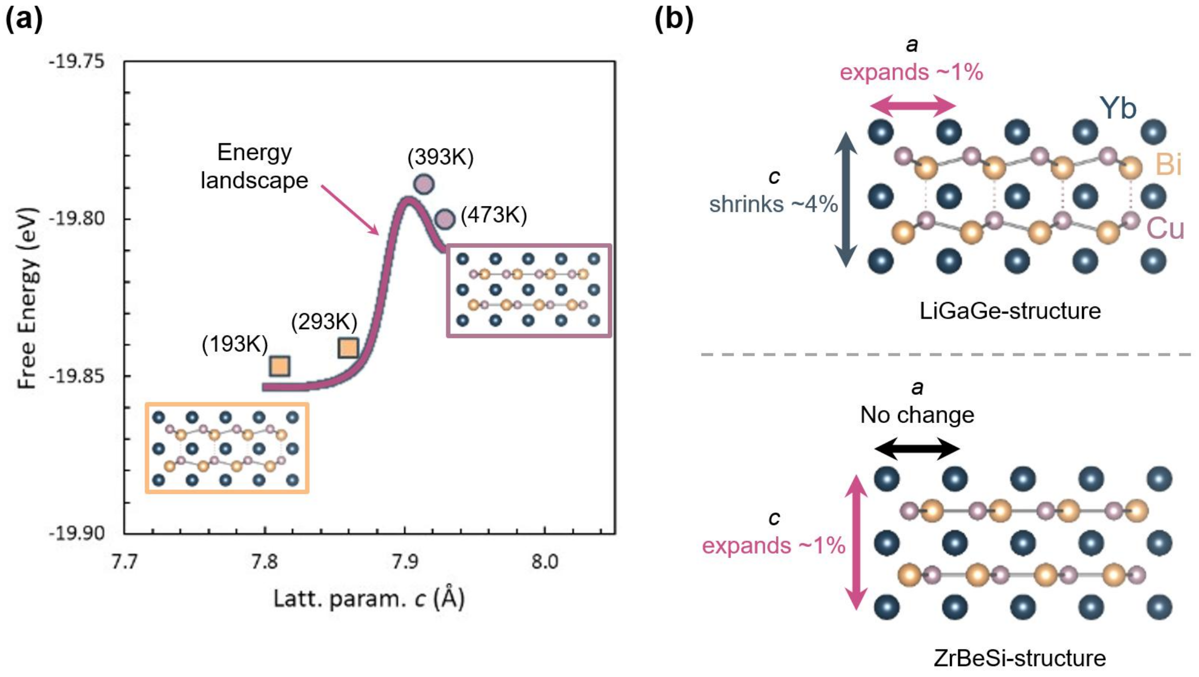

Figure S1: (a) Single point energy calculations reveal an energy barrier between the two structures. The crystal structures are taken from Tkachuk *et al.* (b) Structure relaxation pushes the out-of-plane lattice parameters in opposite directions for the two YbCuBi structures, likely due to the interlayer interactions.

Table S1. Lattice parameters and z-position of Cu/Bi from ground state DFT calculations (at 0 K) on the two structural variants of YbCuBi. Both lattice parameters and atomic positions were allowed to be relaxed from the reference values collected from literature[1].

		a_{hex} (Å)	c_{hex} (Å)	*z position*	
Flat YbCuBi ($P6_3/mmc$)	Ref (473 K)	4.59	7.91	Cu	0.250
				Bi	0.750
	DFT	4.59	7.99	Cu	0.250
				Bi	0.750
Corrugated YbCuBi ($P6_3mc$)	Ref (273 K)	4.58	7.81	Cu	0.285
				Bi	0.740
	DFT	4.63	7.55	Cu	0.317
				Bi	0.718

Table S2. The elastic tensor extracted from DFT calculations on the two structural variants of YbCuBi. Young's and shear modulus are calculated using the Voigt-Reuss-Hill approximation. The DFT results are compared to polycrystalline elastic constants extracted from RUS at 300 K.

	c_{11} (GPa)	c_{33} (GPa)	c_{44} (GPa)	c_{12} (GPa)	c_{13} (GPa)	c_{55} (GPa)	Young's modulus (GPa)	Shear modulus (GPa)
Flat YbCuBi ($P6_3/mmc$)	108	51.8	23.2	62.5	21.8	28.4	66.3	26.1
Corrugated YbCuBi ($P6_3mc$)	103	70.8	29.3	45.0	25.6	42.2	83.6	34.1
Corrugated YbCuBi (RUS - 300K)	93.4	-	27.7	-	-	-	71.5	27.7

SI 2. Structure and phase characterization:

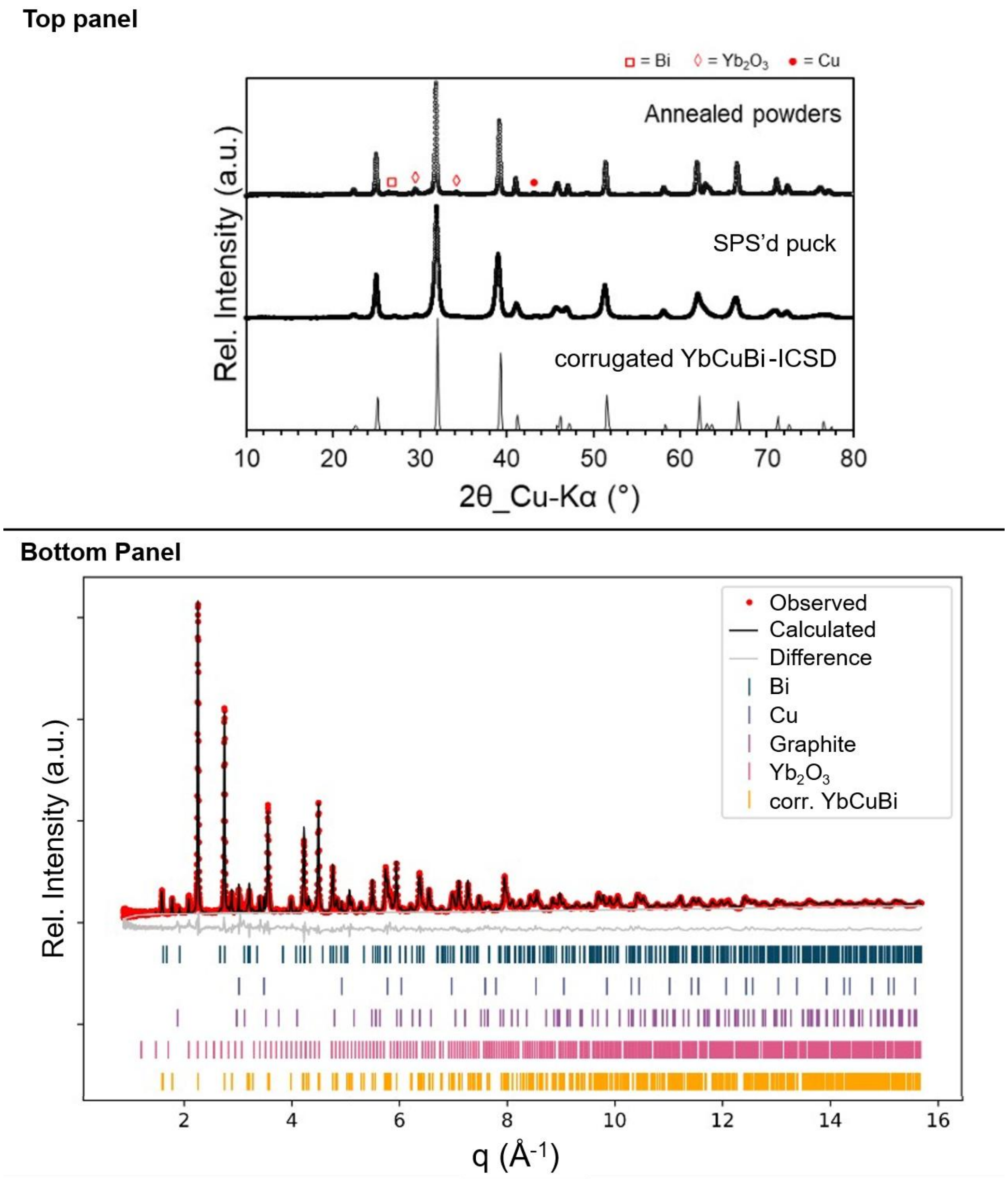

Figure S2: Top panel – XRD patterns collected on SPS'd pucks and subsequently annealed powders show traces of secondary phases. Bottom panel – Rietveld refinement of neutron powder diffraction pattern collected at 30 K was used to quantify the secondary phases. Corrugated quasi-2D YbCuBi accounted for > 90% of the bulk of the sample.

XRD was used to characterize the phases in the sintered disks and subsequently annealed powders (Figure S2 Top panel). After SPS, only the corrugated YbCuBi phase is seen. However, upon grinding into powder and annealing, secondary phases Bi, Cu and Yb_2O_3 become apparent. Traces of graphite were also present on the surface of the sample, as the sample was wrapped in graphite foil before sealing it in the quartz tube for annealing. Annealing also increases crystallinity in the

primary phase by reducing residual stress from ball-milling, which can be inferred from the reduced peak widths. In contrast to x-rays,[2] neutrons have much larger penetration depth[3]. Furthermore, at low temperatures, low thermal vibrations lead to lower atomic displacement parameters. Therefore, neutron powder diffraction (NPD) pattern collected at 30 K was refined to extract the phase composition accurately (SI Figure 2 Bottom Panel).

Neutron powder diffraction patterns were refined for all temperatures to extract the lattice parameters, atomic positions, and atomic displacement parameters (u_{ij}). Lattice parameters show monotonous temperature-dependence and remain unaffected by the crystallographic transition, shown in Figure S3. By symmetry constraints, *x*- and *y*-positions of Bi/Cu atoms are fixed, while a combination of *z*-position and atomic displacement parameter along *z*-direction (represented by u_{33}) differentiates the two crystal structures. The results are shown in the main text Figure 1.

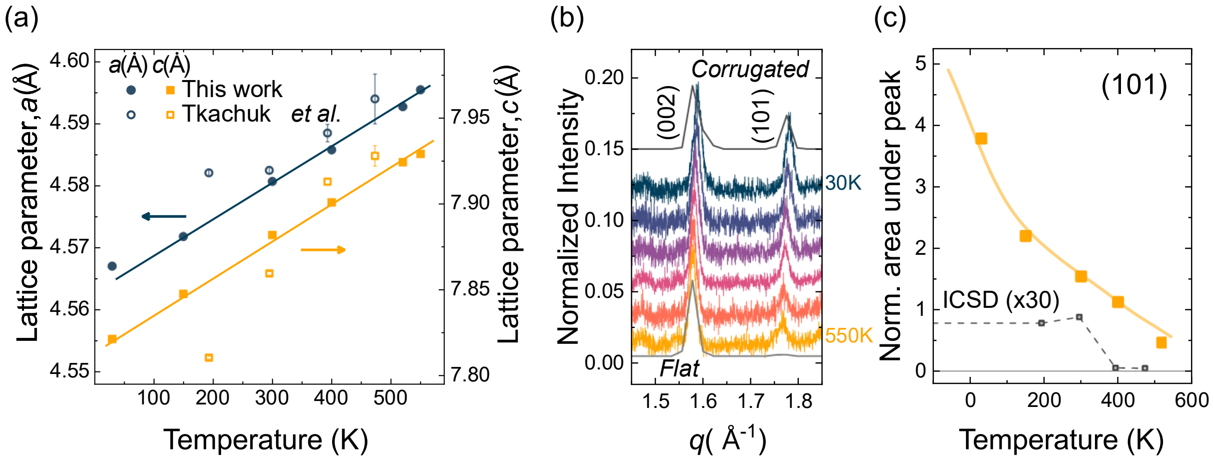

Figure S3: (a) Lattice parameters *a* and *c* are extracted from Rietveld refinement of NPD patterns. Best fit line serves as a guide-to-eye. (b) Zoomed NDP into a narrow region shows the consequences of gained mirror-symmetry, where the (101) peak intensity drops with increasing temperature, but retains a non-zero value. (c) Integrated intensity of the (101) peak, after normalization to maximum intensity, is shown as a function of temperature, which decreases with increasing temperature, reaching a low plateau above 400 K.

SI 3. Specific heat capacity and transport properties: Specific heat capacity, thermal diffusivity, resistivity and thermopower were measured on SPS'd pucks, both on heating and cooling.

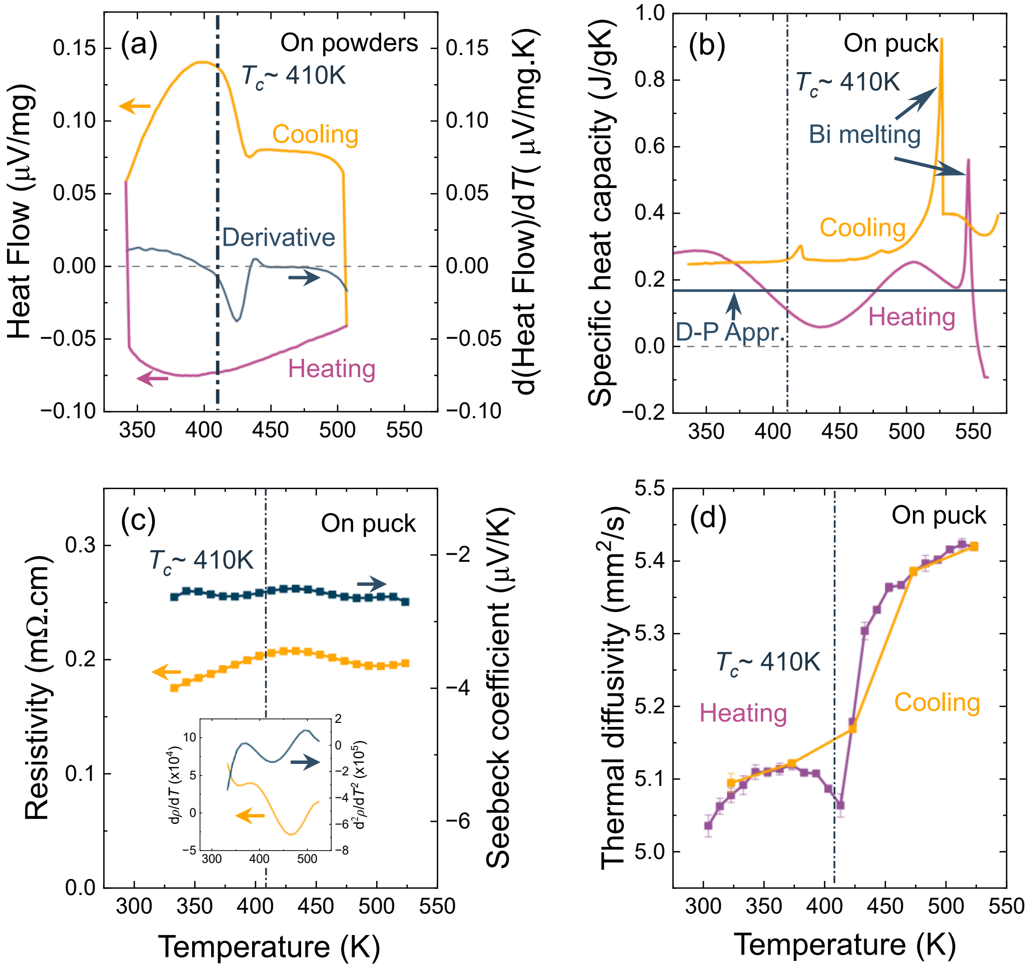

Figure S4: (a) DSC measurements conducted on annealed powders show the phase transition on cooling. (b) Specific heat capacity measurements were conducted using DSC. On heating, the heat capacity fluctuates around the Dulong-Petit approximation for heat capacity close to the phase transition temperature. On cooling, however, the corrugated-to-flat phase transition is picked up as a peak. (c) In contrast, little-to-no change is observed on electronic transport, as both resistivity and Seebeck coefficient remain unaffected by the symmetry change. Resistivity shows broads maxima in first and second derivatives, but the statistical significance is negligible. (d) At the onset temperature from DSC, a step-like increase of 5% is seen in thermal diffusivity, both on heating and cooling.

SI 4. Resonance ultrasound spectroscopy:

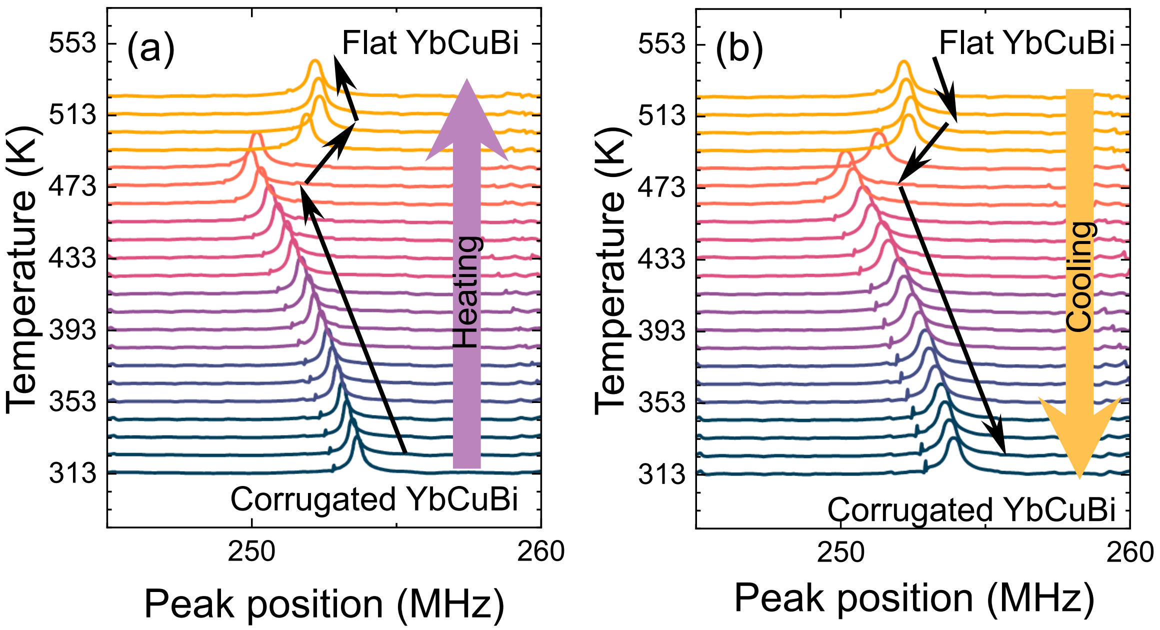

Figure S5: Temperature-dependent evolution of a resonant peak on heating and cooling. At the phase transition, step-like stiffening is observed as the structure changes from

SI 5. Phonon band structure from first-principles DFT:

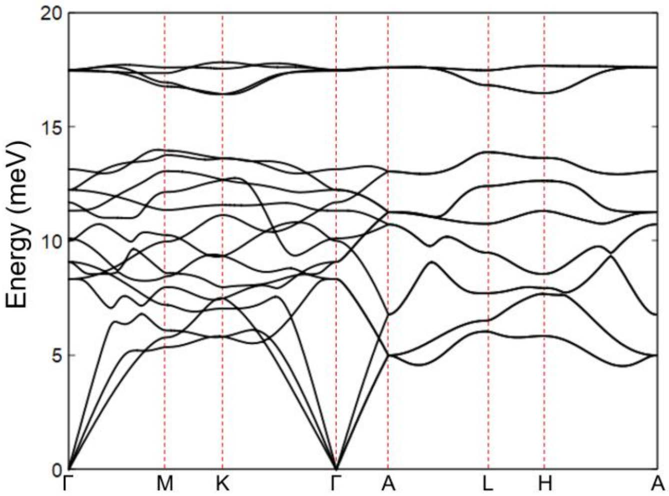

Figure S6. Phonon band structure and density of states calculated from DFT for the corrugated YbCuBi structure (Space group – $P6_3mc$). Imaginary frequency modes were observed in the flat structure, however, suggesting that the flat YbCuBi structure is not stable at 0 K.

SI 6: Reduction and analysis of inelastic neutron scattering spectra:

NW-DOS, $G^{NW}(E)$, can be represented in terms of partial density of states, $g_i(E)$, and their respective neutron-weighting factors, σ_i/m_i, as[4]:

$$G^{NW}(E) = \frac{\sigma_{Yb}}{m_{Yb}} g_{Yb}(E) + \frac{\sigma_{Cu}}{m_{Cu}} g_{Cu}(E) + \frac{\sigma_{Bi}}{m_{Bi}} g_{Bi}(E) \quad \text{(S.1)}$$

Element-specific projection factors from DFT $\left(\frac{g_i^{DFT}(E)}{\sum_i g_i^{DFT}(E)}\right)$ can be used to calculate the partial density of states from experiment using the following equation[4,5]:

$$g_i^{Exp}(E) = \frac{g_i^{DFT}(E).\frac{\sigma_i}{m_i}}{\sum_i \left\{ g_i^{DFT}(E).\frac{\sigma_i}{m_i} \right\}} . G^{NW}(E) \quad \text{(S.2)}$$

Thus-derived true DOS and element-specific partial DOS are shown in Figure S6(a).

Subsequently, force constants of each element (Figure S6(b)) were calculated from partial density of states using the following equation[6]:

$$\langle F \rangle = \frac{M}{\hbar^2} \int_0^\infty g(E). E^2 \, dE \quad \text{(S.3)}$$

Element-specific self-force constants, as extracted from DFT, are shown in Table S2.

Table S3: Anisotropic force constants extracted from DFT for each element from the relaxed corrugated YbCuBi structure (Space group - $P6_3mc$)

	Along *x* (N/m)	Along *z* (N/m)	Average (N/m)
Yb	66.5	68.1	67.1
Cu	60.2	22.3	47.6
Bi	113.8	78.8	102.1

SI 7: Two-mass-two-spring Born-von Karman model:

For two masses m_1 and m_2 connected by two alternating spring constants k_1 and k_2, we can write the equations of motion for each mass in time, *t*, as it interacts with adjacent atoms as[7]:

$$m_1 \frac{d^2 u_s}{dt^2} = k_1 v_s + k_2 v_{s-1} - k_1 u_s - k_2 u_s \tag{S.3}$$

$$m_2 \frac{d^2 v_s}{dt^2} = k_1 u_s + k_2 u_{s+1} - k_1 v_s - k_2 v_s \tag{S.4}$$

where u_s and v_s represent integer displacement (*s*) of atoms m_1 and m_2 respectively, while u_{s+1} represents displacement of atom m_1 in adjacent unit cell. Therefore, the displacements take the traveling wave equation forms:

$$u_s = u\ exp\ exp\ (isKa)\ exp\ (-i\omega t) \tag{S.5}$$

$$v_s = v\ exp\ exp\ (isKa)\ exp\ (-i\omega t) \tag{S.6}$$

where *K* is the wavevector, *a* is the lattice parameter (*c* in this case), and *ω* is the angular frequency. Substituting equations (S.5) and (S.6) into (S.3) and (S.4) yield the following equations:

$$\omega^2 . e_1 = e_1 . \frac{k_1 + k_2}{m_1} - e_2 . \frac{k_1 + k_2 expexp\ (-iKa)}{\sqrt{m_1 m_2}} \tag{S.7}$$

$$\omega^2 . e_2 = e_2 . \frac{k_1 + k_2}{m_2} - e_1 . \frac{k_1 + k_2 expexp\ (-iKa)}{\sqrt{m_1 m_2}} \tag{S.8}$$

where $e_1 = u . \sqrt{m_1}$ and $e_2 = v . \sqrt{m_2}$. These linear equations have a solution only if the determinant of the coefficients of *u* and *v* vanish, and therefore the dynamical matrix of the following form is obtained:

$$D(K) = \begin{vmatrix} \frac{k_1 + k_2}{m_1} & -\left(\frac{k_1 + k_2 expexp\ (-iKa)}{\sqrt{m_1 m_2}}\right) \\ -\left(\frac{k_1 + k_2 expexp\ (-iKa)}{\sqrt{m_1 m_2}}\right) & \frac{k_1 + k_2}{m_2} \end{vmatrix} = 0 \tag{S.9}$$

This model can be applied to the flat and corrugated YbCuBi structures (see main text for details). The dispersion relationships calculated for YbCuBi are shown in Figure S8.

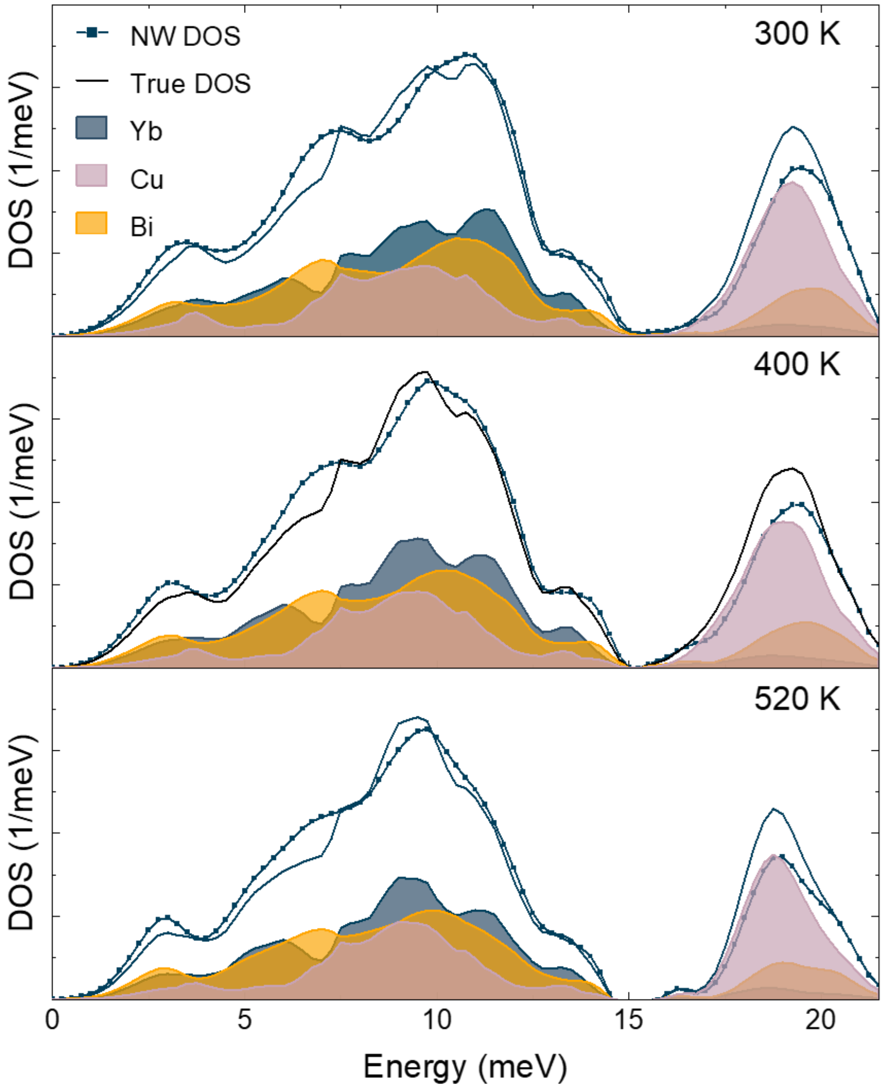

Figure S7. NW-DOS from INS experiments are resolved into element-specific partial DOS and neutron-corrected true DOS at 300 K, 400 K, and 520 K.

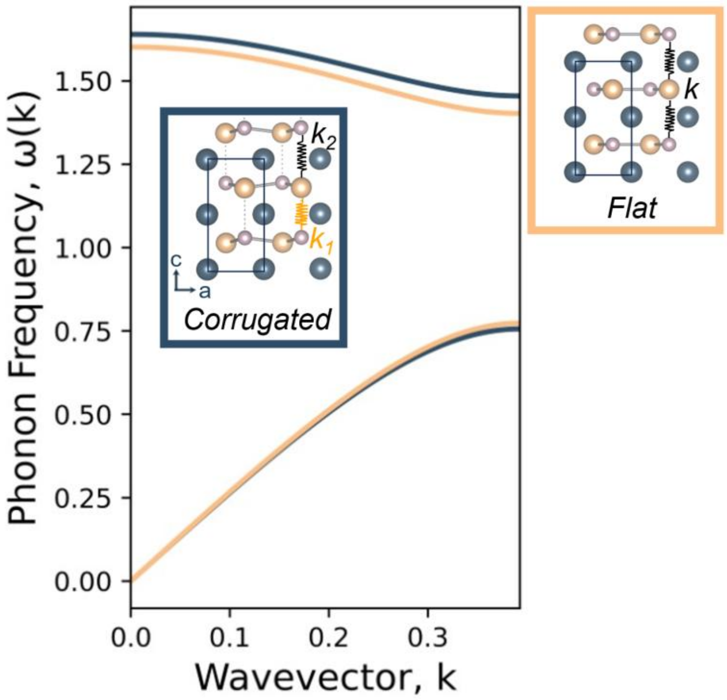

Figure S8: Solving the dynamical matrices using the two-mass-two-spring model for YbCuBi yields two different solutions, shown here. According to the model, simultaneous stiffening of acoustic modes and softening of optical modes can be expected in YbCuBi upon heating.

References

(1) Tkachuk, A.; Mozharivskyj, Y.; Mar, A. Low- and High-Temperature Structures of YbCuBi. *Z. Kristallogr.* 2006, *221*, 539–542. https://doi.org/10.1524/zkri.2006.221.5–7.539.

(2) Finfrock, Y. Z.; Stern, E. A.; Yacoby, Y.; Alkire, R. W.; Evans-Lutterodt, K.; Stein, A.; Isakovic, A. F.; Kas, J. J.; Joachimiak, A. Spatial Dependence and Mitigation of Radiation Damage by a Line-Focus Mini-Beam. *Acta Crystallogr. D* 2010, *66* (12), 1287–1294. https://doi.org/10.1107/S0907444910036875.

(3) Dronskowski, R.; Brückel, T.; Kohlmann, H.; Avdeev, M.; Houben, A.; Meven, M.; Hofmann, M.; Kamiyama, T.; Zobel, M.; Schweika, W.; Hermann, R. P.; Sano-Furukawa, A. Neutron Diffraction: A Primer. *Z Kristallogr Cryst Mater* 2024, *239* (5–6), 139–166. https://doi.org/10.1515/zkri-2024-0001.

(4) Bansal, D.; Hong, J.; Li, C. W.; May, A. F.; Porter, W.; Hu, M. Y.; Abernathy, D. L.; Delaire, O. Phonon Anharmonicity and Negative Thermal Expansion in SnSe. *Phys Rev B* 2016, *94* (5), 1–13. https://doi.org/10.1103/PhysRevB.94.054307.

(5) Pu, Y.; Moseley, D.; He, Z.; Pitike, K. C.; Manley, M. E.; Yan, J.; Cooper, V. R.; Mitchell, V.; Peterson, V. K.; Johannessen, B.; Hermann, R. P.; Cao, P. (Mg,Mn,Fe,Co,Ni)O: A Rocksalt High-Entropy Oxide Containing Divalent Mn and Fe. *Sci Adv* 2023, *9* (38), eadi8809. https://doi.org/10.1126/sciadv.adi8809.

(6) Moseley, D. H.; Thebaud, S. J.; Lindsay, L. R.; Cheng, Y.; Abernathy, D. L.; Manley, M. E.; Hermann, R. P. Temperature-Dependent Lattice Dynamics in Iridium. *Phys Rev Mater* 2020, *4* (11), 113608. https://doi.org/10.1103/PhysRevMaterials.4.113608.

(7) Kittel, C. *Introduction To Solid State Physics*; Wiley: New York, 2019.
